# Development Of The BAJJAJJA Intervention Designed To Meet Grandmother-Caregivers’ Needs In Uganda

**DOI:** 10.1093/geroni/igaf122.2353

**Published:** 2025-12-31

**Authors:** Schola Matovu, Jacqueline Eaton, Lee Ellington, Heather Young

**Affiliations:** University of Utah College of Nursing, Salt Lake City, Utah, United States; University of Utah, Salt Lake City, Utah, United States; University of Utah, Salt Lake City, Utah, United States; Betty Irene Moore School of Nursing at UC Davis, Sacramento, California, United States

## Abstract

The financial, social, health and overall well-being of Ugandan grandmothers who care for their grandchildren has been significantly affected by years of ongoing socioeconomic, political, and historical HIV/AIDS-related challenges. We engaged a Community Advisory Board (CAB) of key community partners to co-develop a culturally responsive Building A Joint Action for JaJJAs (BAJJAJJA) intervention to address the most salient financial, social, and health challenges. Using a community-engaged and qualitative research approach, we worked with a CAB (N = 10) that consisted of grandmother-caregivers, local council leaders, income generating activity experts, and health providers from local health centers in a peri-urban setting of central Uganda. This participatory approach ensured that intervention development aligned with the Uganda National Council for Science and Technology’s priorities for community engagement while also promoting acceptability and sustainability. The developed BAJJAJJA manual details three core intervention components: (1) Income-Generating Activities (IGA)—training in small business skills and cooperative saving practices to enhance financial security; (2) Social Support Groups—peer-led gatherings to strengthen emotional resilience and shared problem-solving; and (3) Health Coaching—education on chronic disease prevention, mental well-being, and self-care strategies with culturally relevant materials tailored for low-literacy populations. This study highlights the effectiveness of a community-engaged approach in designing interventions that address the multidimensional needs of aging grandmother-caregivers. Findings from this study will inform future research that will refine, adapt and test the efficacy of similar interventions in both low-resource and other global settings such as rural areas in the US.

